# On standard conjugate families for natural exponential families with bounded natural parameter space

**DOI:** 10.1016/j.jmva.2014.01.003

**Published:** 2014-04

**Authors:** Kurt Hornik, Bettina Grün

**Affiliations:** a Institute for Statistics and Mathematics, WU Wirtschaftsuniversität Wien, Welthandelsplatz 1, 1020 Vienna, Austria; b Department of Applied Statistics, Johannes Kepler University Linz, Altenbergerstraße 69, 4040 Linz, Austria

**Keywords:** Bayesian analysis, Conjugate prior, Elliptical distribution, Exponential family, Linear posterior expectation, Spherical distribution

## Abstract

Diaconis and Ylvisaker (1979) give necessary conditions for conjugate priors for distributions from the natural exponential family to be proper as well as to have the property of linear posterior expectation of the mean parameter of the family. Their conditions for propriety and linear posterior expectation are also sufficient if the natural parameter space is equal to the set of all d-dimensional real numbers. In this paper their results are extended to characterize when conjugate priors are proper if the natural parameter space is bounded. For the special case where the natural exponential family is through a spherical probability distribution  η, we show that the proper conjugate priors can be characterized by the behavior of the moment generating function of η at the boundary of the natural parameter space, or the second-order tail behavior of η. In addition, we show that if these families are non-regular, then linear posterior expectation never holds. The results for this special case are also extended to natural exponential families through elliptical probability distributions.

## 1 Introduction

Let η be a σ-finite measure on $R^{d}$, and consider the natural exponential family (NEF) F through η, with densities

$$f\left(x|\theta \right)=e^{\theta^{\prime }x-M\left(\theta \right)}$$

with respect to η, where the cumulant generating function M(θ) is defined by

$$e^{M\left(\theta \right)}=\int_{R^{d}}e^{\theta^{\prime }x}\;d\eta \left(x\right)$$

(e.g.,  [1]). Let Θ={θ:M(θ)<∞} be the natural parameter space of F. The family of standard conjugate distributions for F (relative to the natural parameter) has densities

$$\pi \left(\theta |s,\nu \right)\propto e^{s^{\prime }\theta -\nu M\left(\theta \right)}$$

with respect to the Lebesgue measure on Θ (e.g.,  [6]).

Let $J\left(s,\nu \right)=\int_{\Theta }e^{s^{\prime }\theta -\nu M\left(\theta \right)}d\theta$. Then the hyperparameters s and ν giving proper standard conjugate distributions are the ones for which J(s,ν)<∞. For Bayesian inference on θ it is natural to employ priors from the standard conjugate family, and it is important to know when these are proper, or yield proper posteriors.

For regular NEFs (i.e.,  Θ is open) where Θ is non-empty, Diaconis and Ylvisaker [4] show in Theorem 1 that if X, the interior of the convex hull of the support of η, is a non-empty open set in $R^{d}$, then J(s,ν)<∞ if ν>0 and s/ν∈X, and conversely, if $\Theta =R^{d}$, then J(s,ν)<∞ implies that ν>0 and s/ν∈X. (Note that the reference uses νs where we use  s.) This gives a complete characterization of all proper conjugate distributions for the case $\Theta =R^{d}$, leaving open the cases where $\Theta \subset R^{d}$.

In this paper, we prove that if Θ is *bounded*, there exists $-\infty \leq \nu_{0}\leq 0$ such that for arbitrary s, the conjugate priors with hyperparameters ν and s are proper for $\nu >\nu_{0}$ and improper for $\nu <\nu_{0}$. We provide examples showing that all values for $\nu_{0}$ in the range $-\infty \leq \nu_{0}\leq 0$ are possible.

More specific results are obtained when η is a (non-degenerate) spherical probability distribution on $R^{d}$, i.e., a distribution invariant to orthogonal transformations. In this case, X is of the form {x:‖x‖<σ}, where σ is finite if and only if η has bounded support, and Θ is an open or closed ball with radius ρ for some 0<ρ≤∞. For ρ=∞, $\Theta =R^{d}$, and the result of Theorem 1 in Diaconis and Ylvisaker [4] yields that the hyperparameters giving proper conjugate priors are those for which ν>0 and ‖s‖<σν. For ρ<∞, our characterization applies, and we show that lower (and/or upper) bounds for $\nu_{0}$ can be derived if the behavior of the moment generating function of η at the boundary of the natural parameter space can be characterized via asymptotic lower (and/or upper) bound functions. In addition we establish that $\nu_{0}$ can be related to the “second order tail behavior” of η.

If θ∈int(Θ), $\mu \left(\theta \right)=\nabla M\left(\theta \right)=\int_{R^{d}}xf\left(x|\theta \right)d\eta \left(x\right)$ is the mean parameter of the NEF. Diaconis and Ylvisaker [4] show in Theorem 2 that if Θ is open and θ has a distribution which corresponds to a proper conjugate prior with hyperparameters s and ν satisfying s/ν∈X and ν>0, then E(∇M(θ))=s/ν. Clearly, in this case the posterior from an observation x is a conjugate distribution with parameters s+x and ν+1, so that E(∇M(θ)|x)=(s+x)/(ν+1) is linear in x. For NEFs through a spherical probability distribution with bounded Θ, we show that E(∇M(θ)) does not exist for ν≤0 if Θ is open, and exists for all s and ν if Θ is closed, where in this case E(∇M(θ))≠s/ν unless s=0 and ν≠0. Finally, we show that if Θ is closed, linear posterior expectation never holds when using canonical priors.

These results for η a (non-degenerate) spherical probability distribution on $R^{d}$ are extended to the case of elliptical distributions as given in Fang et al. [5, p. 31f]. We show that propriety of conjugate priors is only possible if the matrix in the linear transformation is a square matrix of full rank and that if the natural parameter space is bounded the value of $\nu_{0}$ and the characterization of propriety for $\nu =\nu_{0}$ are the same as for the corresponding spherical probability distribution. Similarly linear posterior expectation only holds in the regular case for ν>0 and never holds in the non-regular case when canonical priors are used.

## 2 General NEFs with bounded natural parameter space

We first establish a general result on the propriety of conjugate priors for NEFs with bounded natural parameter space.

Theorem 1 *Let* η *be a* σ*-finite measure on* $R^{d}$ *. Suppose the natural parameter space* Θ *of the NEF through* η *is bounded and non-empty. Then* ν≥0 *and arbitrary* s *give proper conjugate distributions, and there exists* $\nu_{0}=\nu_{0}\left(\eta \right)$ *with* $-\infty \leq \nu_{0}\leq 0$ *such that for arbitrary* s*, the conjugate distributions with parameters* s *and* ν *are proper for* $\nu >\nu_{0}$*, and improper for* $\nu <\nu_{0}$*.*

Proof If Θ is bounded, then clearly J(s,ν)<∞ if and only if $K\left(\nu \right)=\int_{\Theta }e^{-\nu M\left(\theta \right)}d\theta <\infty$, and $K\left(0\right)=\int_{\Theta }d\theta <\infty$. To establish the theorem, it suffices to show that if $K\left(\nu_{1}\right)<\infty$, then K(ν)<∞ for all $\nu >\nu_{1}$. Now M is convex; the assumptions on Θ are readily seen to imply that M is *proper* in the sense of Rockafellar  [9]. By Corollary 12.1.2 of Rockafellar [9], there are $x\in R^{d}$ and α∈R such that $M\left(\theta \right)\geq x^{\prime }\theta +\alpha$ for all θ (i.e.,  M can be bounded below by a hyperplane). Hence, writing $\gamma =\left|\alpha \right|+\left\|x\right\|\sup_{\theta \in \Theta }\left\|\theta \right\|<\infty$, −M(θ)≤γ for all θ∈Θ.

Now suppose $K\left(\nu_{1}\right)$ is finite and $\nu >\nu_{1}$. Clearly, for all θ∈Θ, $-\nu M\left(\theta \right)=-\nu_{1}M\left(\theta \right)+\left(\nu -\nu_{1}\right)\left(-M\left(\theta \right)\right)\leq -\nu_{1}M\left(\theta \right)+\left(\nu -\nu_{1}\right)\gamma$ so that

$$K\left(\nu \right)\leq \int_{\Theta }e^{-\nu_{1}M\left(\theta \right)}e^{\left(\nu -\nu_{1}\right)\gamma }d\theta =e^{\left(\nu -\nu_{1}\right)\gamma }K\left(\nu_{1}\right)$$

and hence K(ν) is finite as well. Taking $\nu_{0}=\inf\left\{\nu :K\left(\nu \right)<\infty \right\}$, the proof is complete. □

Remark In contrast to the case where the natural parameter space is equal to $R^{d}$, negative values of ν also give proper prior distributions. In this case the parameter ν cannot be interpreted as a prior sample size. Furthermore, the mean for the prior distribution does not necessarily exist as indicated in the example given by Diaconis and Ylvisaker [4, p. 275].

Remark If Θ is not bounded, $J\left(s,\nu_{1}\right)<\infty$ does not necessarily imply that J(s,ν)<∞ for all $\nu \geq \nu_{1}$. This can straightforwardly be seen for $\Theta =R^{d}$, taking, e.g.,  η to have the density with respect to the Lebesgue measure given by $f\left(x\right)\propto e^{-{\left\|x\right\|}^{2}}$ for $\min\left(x\right)≔\min\left(x_{1},\ldots ,x_{d}\right)>1$, and zero otherwise. Then clearly $\Theta =R^{d}$ and X={x:min(x)>1}. By Theorem 1 of Diaconis and Ylvisaker [4], J(s,ν)<∞ if and only if ν>0 and s/ν∈X, or equivalently, if and only if 0<ν<min(s).

As a counterexample for unbounded $\Theta \subset R^{d}$, take d=1 for simplicity (the case of general d can be handled by taking products as above), and let η have density $f\left(x\right)\propto {\left(x-\mu \right)}^{p}e^{-x}$ for x>μ>0 and zero otherwise, where p>−1. Then X=(μ,∞) and

$$e^{M\left(\theta \right)}=\int_{\mu }^{\infty }e^{\theta x}e^{-x}{\left(x-\mu \right)}^{p}dx=e^{\left(\theta -1\right)\mu }\int_{\mu }^{\infty }e^{-\left(1-\theta \right)\left(x-\mu \right)}{\left(x-\mu \right)}^{p}dx=e^{\left(\theta -1\right)\mu }\int_{0}^{\infty }e^{-\left(1-\theta \right)t}t^{p}dt=e^{\left(\theta -1\right)\mu }\frac{\Gamma \left(p+1\right)}{{\left(1-\theta \right)}^{p+1}}$$

for θ<1, so that Θ=(−∞,1). Thus,

$$J\left(s,\nu \right)=\frac{1}{\Gamma {\left(p+1\right)}^{\nu }}\int_{-\infty }^{1}e^{s\theta }{\left(1-\theta \right)}^{\left(p+1\right)\nu }e^{-\nu \mu \left(\theta -1\right)}d\theta \text{.}$$

For convergence, we need (p+1)ν>−1 (otherwise the singularity at θ=1 integrates to infinity), i.e.,  $\nu >-1/\left(p+1\right)=\nu_{0}\left(p\right)$, and s>νμ, or equivalently, $\nu_{0}\left(p\right)<\nu <s/\mu$. Note that $p\mapsto \nu_{0}\left(p\right)$ is increasing for p>−1 with $\lim_{p\to -1+}\nu_{0}\left(p\right)=-\infty$ and $\lim_{p\to \infty }\nu_{0}\left(p\right)=0$. Hence, by suitably choosing p, we can achieve that for arbitrary negative $\nu_{0}$ we have proper priors for hyperparameters satisfying $\nu_{0}<\nu <s/\mu$ (containing the range where ν>0 and s/ν∈X, i.e.,  ν<s/μ, as a proper subset).

## 3 NEFs through spherical probability distributions

In what follows, we restrict our attention to natural exponential families through spherical probability distributions.

Suppose that η is an orthogonally invariant probability measure on $R^{d}$. Then if X is distributed according to η, it has a representation $X{=}^{d}RU$ where U is uniformly distributed on the unit hypersphere $S^{d-1}$, R is a non-negative scalar, and R and U are independent (e.g.,  [5], p. 30). Equivalently, if we consider the polar decomposition X=‖X‖U, its polar part U is uniform, and independent of ‖X‖. Write $\eta_{R}$ for the probability measure on [0,∞) with distribution function $F_{\eta_{R}}\left(r\right)=\eta \left(\left\{x:\left\|x\right\|\leq r\right\}\right)$, and $\eta_{U}$ for the uniform distribution on $S^{d-1}$. Following [5], we will say that η is the spherical probability distribution generated by $\eta_{R}$.

Clearly, if r is in the support of $\eta_{R}$, then all points on the hypersphere with radius r are in the support of η. Hence, if we write $\sigma =\sup\left\{r:F_{\eta_{R}}\left(r\right)<1\right\}$ for the supremum of the support of $F_{\eta_{R}}$, the interior X of the convex hull of the support of η is the open ball with radius σ (and hence $R^{d}$ if $\eta_{R}$ has unbounded support).

Using the fact that

$$\int_{S^{d-1}}e^{\gamma^{\prime }u}\;d\eta_{U}\left(u\right)={}_{0}F_{1}\left(;d/2;{\left\|\gamma \right\|}^{2}/4\right)\text{,}$$

where

$${}_{0}F_{1}\left(;\nu ;z\right)=\overset{\infty }{\underset{n=0}{\sum }}\frac{\Gamma \left(\nu \right)}{\Gamma \left(\nu +n\right)}\frac{z^{n}}{n!}$$

is a generalized hypergeometric series (e.g.,  [7], p. 168, by rewriting the modified Bessel function $I_{\nu }$ in terms of ${}_{0}F_{1}$) we obtain that the moment generating function of η satisfies

$$\int_{R^{d}}e^{\theta^{\prime }x}\;d\eta \left(x\right)=\int_{0}^{\infty }\int_{S^{d-1}}e^{r\theta^{\prime }u}\;d\eta_{U}\left(u\right)\;d\eta_{R}\left(r\right)=\int_{0}^{\infty }{}_{0}F_{1}\left(;d/2;r^{2}{\left\|\theta \right\|}^{2}/4\right)\;d\eta_{R}\left(r\right)=\int_{0}^{\infty }\left(\overset{\infty }{\underset{n=0}{\sum }}\frac{\Gamma \left(d/2\right)}{\Gamma \left(d/2+n\right)}\frac{{\left(r\left\|\theta \right\|\right)}^{2n}}{4^{n}n!}\right)\;d\eta_{R}\left(r\right)=\overset{\infty }{\underset{n=0}{\sum }}c_{n}m_{2n}{\left\|\theta \right\|}^{2n}\text{,}$$

where

$$c_{n}=\frac{\Gamma \left(d/2\right)}{\Gamma \left(d/2+n\right)}\frac{1}{4^{n}n!}\text{,}\;m_{2n}=\int_{0}^{\infty }r^{2n}\;d\eta_{R}\left(r\right)\text{,}$$

so that if d=1, $c_{n}=1/\left(2n\right)!$; if d=2, $c_{n}=1/\left(4^{n}{\left(n!\right)}^{2}\right)$; if d=3, $c_{n}=1/\left(2n+1\right)!$.

Thus, the moment generating function of η is a function of the maximal invariant function ${\left\|\theta \right\|}^{2}$ for the group of orthogonal transformations acting on $R^{d}$, and (where finite) admits a power series representation with coefficients given by the moments of $\eta_{R}$. (Clearly, $\int_{R^{d}}e^{\theta^{\prime }x}d\eta \left(x\right)$ can be finite for θ≠0 only if all moments of $\eta_{R}$ are finite.)

Let

$$c\left(z\right)=\overset{\infty }{\underset{n=0}{\sum }}c_{n}m_{2n}z^{n}$$

so that $e^{M\left(\theta \right)}=c\left({\left\|\theta \right\|}^{2}\right)$. Note that for r≥0, $r\mapsto c\left(r^{2}\right)$ is non-decreasing with $c\left(0\right)=c_{0}m_{0}=1$. Let $\rho^{2}$ be the radius of convergence of c(z). Then clearly, if $c\left(\rho^{2}-\right)=\infty$, Θ is the open ball with radius ρ, and the NEF is regular; otherwise, Θ is the closed ball with radius ρ. In analogy to the notion of the characteristic generator (for which the characteristic function at θ equals $\phi \left({\left\|\theta \right\|}^{2}\right)$) employed by Fang et al. [5], we will refer to c as the *moment generator* of η.

### 3.1 Propriety of canonical priors

For spherical distributions where the parameter space Θ is bounded Theorem 1 is applicable, but does not provide any insight on specific values of $\nu_{0}$. In the following we use the familiar Bachmann–Landau notations defined in the following way:

$$f\left(x\right)=O\left(g\left(x\right)\right)\Leftrightarrow \exists C>0:\underset{x\to a}{\lim}\left|\frac{f\left(x\right)}{g\left(x\right)}\right|\leq C$$

$$f\left(x\right)=\Omega \left(g\left(x\right)\right)\Leftrightarrow \exists C>0:\underset{x\to a}{\lim}\left|\frac{f\left(x\right)}{g\left(x\right)}\right|\geq C$$

$$f\left(x\right)≍g\left(x\right)\Leftrightarrow \exists C_{u},C_{l}>0:\underset{x\to a}{\lim}\left|\frac{f\left(x\right)}{g\left(x\right)}\right|\leq C_{u},\underset{x\to a}{\lim}\left|\frac{f\left(x\right)}{g\left(x\right)}\right|\geq C_{l}$$

$$f\left(x\right)=o\left(g\left(x\right)\right)\Leftrightarrow \forall C>0:\underset{x\to a}{\lim}\left|\frac{f\left(x\right)}{g\left(x\right)}\right|\leq C\text{.}$$

Using this notation the following theorem provides a characterization of $\nu_{0}\left(\eta \right)$ for spherical η in terms of the behavior of the moment generator of η at its convergence radius.

Theorem 2 *Let* η *be a spherical probability distribution on* $R^{d}$ *with moment generator* c *and* $\rho^{2}$*the radius of convergence of* c *. Suppose that* 0<ρ<∞ *. Let* β>0*.*

(a) *If* $\lim_{r\to \rho -}c\left(r^{2}\right)$ *is finite,* $\nu_{0}\left(\eta \right)=-\infty$*.*
(b) *If* $c\left(r^{2}\right)=O\left({\left(\rho -r\right)}^{-\beta }\right)$ *as* r→ρ−*,* $\nu_{0}\left(\eta \right)\leq -1/\beta$*.*
(c) *If* $c\left(r^{2}\right)=\Omega \left({\left(\rho -r\right)}^{-\beta }\right)$ *as* r→ρ−*,* $\nu_{0}\left(\eta \right)\geq -1/\beta$*.*
(d) *If* $c\left(r^{2}\right)≍{\left(\rho -r\right)}^{-\beta }$ *as* r→ρ−*,* $\nu_{0}\left(\eta \right)=-1/\beta$*.*

Proof If the moment generator c has radius of convergence equal to $\rho^{2}$ with 0<ρ<∞, then the natural parameter space Θ of the NEF through η satisfies {θ:‖θ‖<ρ}⊆Θ⊆{θ:‖θ‖≤ρ} and hence is bounded and non-empty.

Using the polar decomposition θ=ru, $d\theta =a_{d}r^{d-1}drd\eta_{U}\left(u\right)$, where $a_{d}$ is the area of $S^{d-1}$,

$$K\left(\nu \right)=\int_{\Theta }e^{-\nu M\left(\theta \right)}d\theta =\int_{0}^{\rho }\int_{S^{d-1}}c{\left(r^{2}\right)}^{-\nu }a_{d}r^{d-1}drd\eta_{U}\left(u\right)=a_{d}\int_{0}^{\rho }r^{d-1}c{\left(r^{2}\right)}^{-\nu }\int_{S^{d-1}}d\eta_{U}\left(u\right)dr=a_{d}\int_{0}^{\rho }r^{d-1}c{\left(r^{2}\right)}^{-\nu }dr\text{.}$$

By Theorem 1, K(ν) is finite for ν≥0. Hence, suppose ν<0. As $r\mapsto c\left(r^{2}\right)$ is non-decreasing on [0,ρ) with c(0)=1, K(ν) is finite if and only if $K_{\epsilon }\left(\nu \right)=\int_{\rho -\epsilon }^{\rho }c{\left(r^{2}\right)}^{-\nu }dr$ is finite for some ϵ>0. This implies assertion (a), i.e., if $\lim_{r\to \rho -}c\left(r^{2}\right)$ is finite, $K_{\epsilon }$ is finite for all ν.

Let β>0. If $c\left(r^{2}\right)$ is ≤ (or ≥) $C{\left(\rho -r\right)}^{-\beta }$ on (ρ−ϵ,ρ) for positive ϵ and C, −ν>0 implies that $K_{\epsilon }\left(\nu \right)$ is ≤ (or ≥)

$$\int_{\rho -\epsilon }^{\rho }{\left(C{\left(\rho -r\right)}^{-\beta }\right)}^{-\nu }dr=C^{-\nu }\int_{0}^{\epsilon }r^{\nu \beta }dr\text{,}$$

respectively, which converges if and only if νβ>−1, or equivalently, if and only if ν>−1/β. Thus, if $c\left(r^{2}\right)=O\left({\left(\rho -r\right)}^{-\beta }\right)$ as r→ρ−, K(ν) is finite for all ν>−1/β, and hence $\nu_{0}\left(\eta \right)\leq -1/\beta$ (assertion (b)). Conversely, if $c\left(r^{2}\right)=\Omega \left({\left(\rho -r\right)}^{-\beta }\right)$ as r→ρ−, K(ν) is infinite for all ν≤−1/β, and hence $\nu_{0}\left(\eta \right)\geq -1/\beta$ (assertion (b)). Assertion (d) follows by combining these two results. □

Our next result shows how for spherical η, $\nu_{0}\left(\eta \right)$ can also be characterized in terms of the tail behavior of the generating distribution $\eta_{R}$.

Theorem 3 *Let* η *be a spherical probability distribution on* $R^{d}$ *such that* $\eta_{R}\left(\left(r,\infty \right)\right)=\eta \left(\left\{x:\left\|x\right\|>r\right\}\right)≍e^{-\rho r}r^{\delta }$ *as* r→∞*, for some* ρ>0 *and* δ∈R *. Then the natural parameter space* Θ *of the NEF through* η *satisfies* {θ:‖θ‖<ρ}⊆Θ⊆{θ:‖θ‖≤ρ} *and hence is bounded and non-empty, and prior distributions from the standard conjugate family are proper for all* $s\in R^{d}$ *and* ν∈R *if* δ≤(d−3)/2 *(corresponding to* $\nu_{0}\left(\eta \right)=-\infty$ *), and for all* $s\in R^{d}$ *and* $\nu >\nu_{0}\left(\eta \right)=1/\left(\left(d-3\right)/2-\delta \right)$ *if* δ>(d−3)/2*.*

Proof For x>0,

$$\int_{0}^{\infty }e^{-\rho r}r^{x-1}dr=\int_{0}^{\infty }e^{-u}{\left(u/\rho \right)}^{x-1}du/\rho =\Gamma \left(x\right)/\rho^{x}\text{.}$$

For n>0,

$$m_{2n}=\int_{0}^{\infty }s^{2n}d\eta_{R}\left(s\right)=\int_{0}^{\infty }\left(\int_{0}^{\infty }2n1_{\left[0,s\right)}\left(r\right)r^{2n-1}dr\right)d\eta_{R}\left(s\right)=\int_{0}^{\infty }2nr^{2n-1}\left(\int_{0}^{\infty }1_{\left(r,\infty \right)}\left(s\right)d\eta_{R}\left(s\right)\right)dr=2n\int_{0}^{\infty }r^{2n-1}\eta_{R}\left(\left(r,\infty \right)\right)dr\text{,}$$

interchanging the order of integration being justified by Fubini’s theorem.

By assumption, there exist positive and finite constants $C_{l}$ and $C_{u}$ such that for all r sufficiently large,

$$0<C_{l}\leq \frac{\eta_{R}\left(\left(r,\infty \right)\right)}{e^{-\rho r}r^{\delta }}\leq C_{u}<\infty \text{.}$$

By possibly modifying these constants, we can in fact assume that these inequalities hold for all r≥1. Then for all n>0 such that 2n+δ>0,

$$m_{2n}\leq 2n\int_{0}^{1}r^{2n-1}dr+2n\int_{1}^{\infty }r^{2n-1}C_{u}e^{-\rho r}r^{\delta }dr\leq 1+2nC_{u}\int_{0}^{\infty }e^{-\rho r}r^{2n+\delta -1}dr=1+C_{u}\frac{2n}{2n+\delta }\left(2n+\delta \right)\frac{\Gamma \left(2n+\delta \right)}{\rho^{2n+\delta }}=1+C_{u}\frac{2n}{2n+\delta }\frac{\Gamma \left(2n+\delta +1\right)}{\rho^{2n+\delta }}\text{.}$$

Thus, there is a $C_{u}^{*}<\infty$ such that for all n sufficiently large,

$$m_{2n}\leq C_{u}^{*}\frac{\Gamma \left(2n+\delta +1\right)}{\rho^{2n+\delta }}\text{.}$$

Conversely,

$$m_{2n}\geq 2n\int_{1}^{\infty }r^{2n-1}C_{l}e^{-\rho r}r^{\delta }dr=2nC_{l}\left(\int_{0}^{\infty }e^{-\rho r}r^{2n+\delta -1}dr-\int_{0}^{1}e^{-\rho r}r^{2n+\delta -1}dr\right)\geq 2nC_{l}\frac{\Gamma \left(2n+\delta \right)}{\rho^{2n+\delta }}-2nC_{l}\int_{0}^{1}r^{2n+\delta -1}dr=C_{l}\frac{2n}{2n+\delta }\frac{\Gamma \left(2n+\delta +1\right)}{\rho^{2n+\delta }}-C_{l}\frac{2n}{2n+\delta }\text{.}$$

Thus, there is $C_{l}^{*}>0$ such that for all n sufficiently large,

$$m_{2n}\geq C_{l}^{*}\frac{\Gamma \left(2n+\delta +1\right)}{\rho^{2n+\delta }}\text{.}$$

Therefore, $m_{2n}≍\Gamma \left(2n+\delta +1\right)/\rho^{2n}$ as n→∞, and the moment generator $c\left(z\right)=\sum_{n}c_{n}m_{2n}z^{n}$ has the same convergence radius as $\overset{˜}{c}\left(z\right)=\sum_{n}c_{n}\Gamma \left(2n+\delta +1\right){\left(z/\rho^{2}\right)}^{n}$, and the same asymptotic behavior as z converges to the convergence radius from below.

Using Pochhammer’s symbol ${\left(x\right)}_{n}=\Gamma \left(x+n\right)/\Gamma \left(x\right)$,

$$c_{n}=\frac{\Gamma \left(d/2\right)}{\Gamma \left(d/2+n\right)}\frac{1}{4^{n}n!}=\frac{1}{{\left(d/2\right)}_{n}4^{n}n!}$$

and for δ≥−1,

$$\frac{\Gamma \left(2n+\delta +1\right)}{\Gamma \left(\delta +1\right)}=\overset{n-1}{\underset{i=0}{\prod }}\left(\delta +1+2i\right)\overset{n-1}{\underset{i=0}{\prod }}\left(\delta +2+2i\right)=4^{n}\overset{n-1}{\underset{i=0}{\prod }}\left(\left(\delta +1\right)/2+i\right)\overset{n-1}{\underset{i=0}{\prod }}\left(\left(\delta /2+1\right)+i\right)=4^{n}\frac{\Gamma \left(\left(\delta +1\right)/2+n\right)}{\Gamma \left(\left(\delta +1\right)/2\right)}\frac{\Gamma \left(\left(\delta /2+1\right)+n\right)}{\Gamma \left(\delta /2+1\right)}=4^{n}{\left(\left(\delta +1\right)/2\right)}_{n}{\left(\delta /2+1\right)}_{n}\text{.}$$

Thus,

$$\overset{˜}{c}\left(z\right)=\Gamma \left(\delta +1\right)\underset{n}{\sum }\frac{{\left(\left(\delta +1\right)/2\right)}_{n}{\left(\delta /2+1\right)}_{n}}{{\left(d/2\right)}_{n}n!}{\left(\frac{z}{\rho^{2}}\right)}^{n}=\Gamma \left(\delta +1\right){}_{2}F_{1}\left(\left(\delta +1\right)/2,\delta /2+1;d/2;z/\rho^{2}\right)\text{,}$$

where ${}_{2}F_{1}$ is the Gaussian (or ordinary) hypergeometric function. From well-known results on such functions, the radius of convergence $\overset{˜}{c}$ equals $\rho^{2}$. This implies that the natural parameter space Θ of the NEF through η satisfies {θ:‖θ‖<ρ}⊆Θ⊆{θ:‖θ‖≤ρ} and hence is bounded and non-empty.

The behavior of $\overset{˜}{c}\left(z\right)$ at $\rho^{2}$ and therefore also of c(z) can be obtained, e.g., from Olver et al. [8], Section 15.4(ii), http://dlmf.nist.gov/15.4.ii. We have the following three cases.

– If 0<d/2−(δ+1)/2−(δ/2+1)=(d−2δ−3)/2, or equivalently, δ<(d−3)/2, $\lim_{r\to \rho -}\overset{˜}{c}\left(r^{2}\right)$ exists. In this case, Theorem 2 implies that $\nu_{0}\left(\eta \right)=-\infty$.
– If δ>(d−3)/2, $\overset{˜}{c}\left(r^{2}\right)∼{\left(1-\left(r/\rho \right)\right)}^{\left(d-2\delta -3\right)/2}$ as r→ρ−. We can thus use Theorem 2 with β=−(d−2δ−3)/2 to obtain $\nu_{0}\left(\eta \right)=-1/\beta =2/\left(d-2\delta -3\right)$.
– Finally, if δ=(d−3)/2, $\overset{˜}{c}\left(r^{2}\right)∼-\log\left(1-\left(r/\rho \right)\right)$ as r→ρ−, and Theorem 2 cannot be applied directly. From the proof of Theorem 2, clearly, K(ν)<∞ if and only if $\int_{1/2}^{1}{\left(-\log\left(1-r\right)\right)}^{-\nu }dr$ is finite. Substituting −log(1−r)=u so that $r=1-e^{-u}$,
  $$\int_{1/2}^{1}{\left(-\log\left(1-r\right)\right)}^{-\nu }dr=\int_{\log\left(2\right)}^{\infty }u^{-\nu }e^{-u}du$$
  which again is finite for all ν. Hence, $\nu_{0}\left(\eta \right)=-\infty$.

The above assumed that δ≥−1. Otherwise, for 0≤r<ρ, $\overset{˜}{c}\left(r^{2}\right)$ is majorized by the series for δ=−1, which always has a finite limit as r→ρ−. Thus δ<−1 gives $\nu_{0}\left(\eta \right)=-\infty$, and the proof of the theorem is complete. □

In the following examples different distributions for $\eta_{R}$ are considered, with the Poisson and the negative binomial distributions as examples for discrete distributions and the Gamma distribution as an example for a continuous distribution. For these distributions it is investigated for which parameter values s and ν prior distributions from the standard conjugate family are proper using either Theorem 1 in Diaconis and Ylvisaker [4] or Theorem 3.

Example Suppose $\eta_{R}$ is a Poisson distribution with mean parameter λ. Then

$$\eta_{R}\left(\left(r,\infty \right)\right)=1-\overset{a}{\underset{k=0}{\sum }}\frac{\lambda^{k}}{k!}e^{-\lambda }=\frac{1}{\Gamma \left(a+1\right)}\int_{0}^{\lambda }t^{a}e^{-t}dt\text{,}$$

where a=⌊r⌋. As in the integrand, $e^{-\lambda }\leq e^{-t}\leq 1$ for all 0≤t≤λ, it holds that

$$\eta_{R}\left(\left(r,\infty \right)\right)∼\frac{1}{\Gamma \left(a+1\right)}\int_{0}^{\lambda }t^{a}dt=\frac{\lambda^{a+1}}{\Gamma \left(a+2\right)}=\exp\left(-a\left(\log\left(a\right)+O\left(1\right)\right)\right)$$

as a→∞. Hence, in this case ρ=∞ and Theorem 1 in Diaconis and Ylvisaker  [4] can be used to conclude that the prior distributions from the standard conjugate family are proper if and only if ν>0.

Example Suppose $\eta_{R}$ is a negative binomial distribution with size parameter n and success probability p, where the negative binomial distributed random variable indicates the number of failures before the first success. Then

$$\eta_{R}\left(\left(r,\infty \right)\right)=\frac{1}{B\left(a+1,n\right)}\int_{0}^{p}t^{a}{\left(1-t\right)}^{n-1}dt\text{,}$$

where a=⌊r⌋ and B(a+1,n) is the Beta function. As in the integrand, ${\left(1-p\right)}^{n-1}\leq {\left(1-t\right)}^{n-1}\leq 1$ for all 0≤t≤p, it holds that

ηR((r,∞))∼1B(a+1,n)∫0ptadt=a+nn−1pa+1∼paan−1

as a→∞. Thus, Theorem 3 can be applied with ρ=log(p) and δ=n−1 to obtain that $\nu_{0}\left(\eta \right)=-\infty$ if n≤(d−1)/2, and $\nu_{0}\left(\eta \right)=1/\left(\left(d-1\right)/2-n\right)$ otherwise.

Example Suppose $\eta_{R}$ is a Gamma distribution with shape parameter α and rate parameter ρ. Then

$$\eta_{R}\left(\left(r,\infty \right)\right)=\int_{r}^{\infty }\frac{\rho^{\alpha }}{\Gamma \left(\alpha \right)}e^{-\rho s}s^{\alpha -1}ds=\int_{\rho r}^{\infty }\frac{e^{-u}u^{\alpha -1}}{\Gamma \left(\alpha \right)}du∼\frac{e^{-\rho r}{\left(\rho r\right)}^{\alpha -1}}{\Gamma \left(\alpha \right)}$$

as r→∞ (see for example Olver et al. [8], Section 8.11(i), http://dlmf.nist.gov/8.11.i). Thus, Theorem 3 can be applied with ρ and δ=α−1 to obtain that $\nu_{0}\left(\eta \right)=-\infty$ if α≤(d−1)/2, and $\nu_{0}\left(\eta \right)=1/\left(\left(d-1\right)/2-\alpha \right)$ otherwise. If $\eta_{R}$ is a shape mixture of Gamma distributions with fixed rate parameter ρ, where the mixture density is not compactly supported, the convergence radius is still ρ, but $\nu_{0}\left(\eta \right)$ must satisfy $\nu_{0}>1/\left(\left(d-1\right)/2-\alpha \right)$ for all α sufficiently large, so that $\nu_{0}\left(\eta \right)=0$.

Remark The example where $\eta_{R}$ is a shape mixture of Gamma distributions shows that in fact all values of $\nu_{0}$ in the range [−∞,0] are possible. In fact, this can also be inferred directly from Theorem 3 by fixing $r_{0}\geq 0$ and taking $\eta_{R}$ supported on $\left(r_{0},\infty \right)$ with $\eta_{R}\left(\left(r,\infty \right)\right)\propto e^{-\rho r}r^{\delta }$ for $r\geq r_{0}$ (δ<−1 needs $r_{0}>0$). This again gives $\nu_{0}$ values which monotonically cover the range [−∞,0) as δ varies from −∞ to ∞; $\nu_{0}=0$ can be obtained by using tails which are ≍exp(−ρr(1+o(1))) as r→∞ but are heavier than $e^{-\rho r}r^{\delta }$ for all δ, such as tails proportional to $\exp\left(-\rho r+\delta \sqrt{r}\right)$.

Combining the example where $\eta_{R}$ is a shape mixture of Gamma distributions and the above remark we obtain the following.

Corollary 1 *For every* $\nu_{0}$ *with* $-\infty \leq \nu_{0}\leq 0$ *a* σ*-finite measure* η *on* $R^{d}$ *can be found, for which the natural parameter space* Θ *of the NEF through* η *is bounded and non-empty, and for which the conjugate priors are proper for hyperparameters* $\nu >\nu_{0}$ *and arbitrary* s *and improper for hyperparameters* $\nu <\nu_{0}$ *and arbitrary* s*.*

Remark It would certainly be interesting to investigate in more detail whether and how $\nu_{0}\left(\eta \right)$ can be related to the tail behavior of η in the general (non-spherical) case of bounded Θ covered by Theorem 1 (assuming for example that η has a sufficiently nice density). We leave this for future research.

### 3.2 Linearity of posterior expectation

Next, we investigate how Theorem 2 in Diaconis and Ylvisaker  [4] can be extended in the spherical case where regularity of the NEF is not necessarily given and ν is allowed to be negative.

Theorem 4 *Let* η *be a spherical probability distribution on* $R^{d}$ *with moment generator* c*, and* $0<\rho^{2}<\infty$*the radius of convergence of* c *. Suppose* θ *follows a proper conjugate prior with parameters* s *and* ν *for the NEF through* η*.*

(a) *If* $c\left(\rho^{2}-\right)=\infty$ *(regular case),* E(∇M(θ))=s/ν *for all* s *and* ν>0 *. If* ν≤0*, the expectation does not exist.*
(b) *If* $c\left(\rho^{2}-\right)<\infty$ *(non-regular case), then*
  $$E\left(\nabla M\left(\theta \right)\right)=\left\{ \begin{array}{cc} \frac{s}{\nu }\left(1-\frac{c{\left(\rho^{2}-\right)}^{-\nu }J\left(s,0\right)}{J\left(s,\nu \right)}\right) & \text{if }\nu \neq 0,s\in R^{d}\text{,}\\ \frac{s}{J\left(s,0\right)}\int_{\Theta }\log\left(\frac{c\left({\left\|\theta \right\|}^{2}\right)}{c\left(\rho^{2}-\right)}\right)e^{s^{\prime }\theta }d\theta & \text{if }\nu =0,s\in R^{d}\text{.} \end{array}\right.$$

Proof We have

$$E\left(\nabla M\left(\theta \right)\right)=\frac{1}{J\left(s,\nu \right)}\int_{\Theta }\nabla M\left(\theta \right)e^{s^{\prime }\theta -\nu M\left(\theta \right)}d\theta ≕E\left(s,\nu \right)\text{.}$$

In the spherical case, we have $M\left(\theta \right)=\log\left(c\left({\left\|\theta \right\|}^{2}\right)\right)$ so that for θ∈int(Θ)

$$\nabla M\left(\theta \right)=2\frac{c^{\prime }\left({\left\|\theta \right\|}^{2}\right)}{c\left({\left\|\theta \right\|}^{2}\right)}\theta$$

and

$$E\left(s,\nu \right)=\frac{2}{J\left(s,\nu \right)}\int_{\Theta }\frac{c^{\prime }\left({\left\|\theta \right\|}^{2}\right)}{c{\left({\left\|\theta \right\|}^{2}\right)}^{1+\nu }}e^{s^{\prime }\theta }\theta d\theta \text{,}$$

where the integrand is a positive scalar function times θ, and can be uniformly bounded away from zero and infinity on ‖θ‖<ρ−ϵ for all ϵ>0. Thus, the integral exists if and only if

$$\int_{\rho -\epsilon \leq \left\|\theta \right\|<\rho }\frac{c^{\prime }\left({\left\|\theta \right\|}^{2}\right)}{c{\left({\left\|\theta \right\|}^{2}\right)}^{1+\nu }}\left\|\theta \right\|e^{s^{\prime }\theta }d\theta <\infty \text{,}$$

which upon transforming to polar coordinates is easily seen to be equivalent to

$$\int_{\rho -\epsilon }^{\rho }\frac{c^{\prime }\left(\kappa^{2}\right)}{c{\left(\kappa^{2}\right)}^{1+\nu }}2\kappa d\kappa <\infty \text{.}$$

But the last integral is just $c{\left(\kappa^{2}\right)}^{-\nu }/\left(-\nu \right){|}_{\rho -\epsilon }^{\rho -}$ for ν≠0 and $\log\left(c\left(\kappa^{2}\right)\right){|}_{\rho -\epsilon }^{\rho -}$ for ν=0, and hence infinite if and only if $c\left(\rho^{2}-\right)=\infty$ and ν≤0. Hence, E(∇M(θ)) exists unless $c\left(\rho^{2}-\right)=\infty$ and ν≤0.

To actually compute E(s,ν) (if it exists), we can use $\nabla \left(e^{s^{\prime }\theta -\nu M\left(\theta \right)}\right)=\left(s-\nu \nabla M\left(\theta \right)\right)e^{s^{\prime }\theta -\nu M\left(\theta \right)}$ for θ∈int(Θ) to obtain that for ν≠0,

$$E\left(s,\nu \right)=\frac{1}{\nu }\left(s-\frac{1}{J\left(s,\nu \right)}\int_{\Theta }\nabla e^{s^{\prime }\theta -\nu M\left(\theta \right)}d\theta \right)\text{,}$$

where $\int_{\Theta }\nabla e^{s^{\prime }\theta -\nu M\left(\theta \right)}d\theta$ exists if and only if E(s,ν) exists. Write $\theta_{i}$ for component  i of θ, and $\theta_{-i}$ for θ without component i. Then

$$\int_{\Theta }\frac{\partial }{\partial \theta_{i}}e^{s^{\prime }\theta -\nu M\left(\theta \right)}d\theta =\int_{\left\|\theta_{-i}\right\|<\rho }\int_{-\sqrt{\rho^{2}-{\left\|\theta_{-i}\right\|}^{2}}}^{\sqrt{\rho^{2}-{\left\|\theta_{-i}\right\|}^{2}}}\frac{\partial }{\partial \theta_{i}}e^{s^{\prime }\theta -\nu M\left(\theta \right)}d\theta_{i}d\theta_{-i}=\int_{\left\|\theta_{-i}\right\|<\rho }e^{s^{\prime }\theta -\nu M\left(\theta \right)}{|}_{-\sqrt{\rho^{2}-{\left\|\theta_{-i}\right\|}^{2}}}^{\sqrt{\rho^{2}-{\left\|\theta_{-i}\right\|}^{2}}}d\theta_{-i}=c{\left(\rho^{2}-\right)}^{-\nu }\int_{\left\|\theta_{-i}\right\|<\rho }e^{s_{-i}^{\prime }\theta_{-i}}\left(e^{s_{i}\sqrt{\rho^{2}-{\left\|\theta_{-i}\right\|}^{2}}}-e^{-s_{i}\sqrt{\rho^{2}-{\left\|\theta_{-i}\right\|}^{2}}}\right)d\theta_{-i}\text{.}$$

If $c\left(\rho^{2}-\right)=\infty$ and ν>0, $c{\left(\rho^{2}-\right)}^{-\nu }=0$ and hence E(s,ν)=s/ν (which could also have been obtained from Theorem 2 in Diaconis and Ylvisaker [4]), establishing (a).

If $c\left(\rho^{2}-\right)<\infty$, we obtain that $\int_{\Theta }\nabla e^{s^{\prime }\theta -\nu M\left(\theta \right)}d\theta =c{\left(\rho^{2}-\right)}^{-\nu }h\left(s\right)$, where h does not depend on ν; hence, $h\left(s\right)=\int_{\Theta }\nabla e^{s^{\prime }\theta }d\theta =s\int_{\Theta }e^{s^{\prime }\theta }d\theta =sJ\left(s,0\right)$, establishing (b) for the case where ν≠0. Finally, E(s,0) can be obtained as $\lim_{\nu \to 0}E\left(s,\nu \right)$, where interchanging integration and taking limits is justified as the above shows that the integrals of the absolute values are uniformly bounded on compact ν intervals. Now with $a=c\left(\rho^{2}-\right)$,

$$E\left(s,\nu \right)=\frac{s}{\nu }\left(1-\frac{a^{-\nu }J\left(s,0\right)}{J\left(s,\nu \right)}\right)=sJ\left(s,0\right)\frac{g\left(s,0\right)-g\left(s,\nu \right)}{\nu }\text{,}$$

where $g\left(s,\nu \right)=a^{-\nu }/J\left(s,\nu \right)$, so that

$$\underset{\nu \to 0}{\lim}E\left(s,\nu \right)=sJ\left(s,0\right)\frac{\partial }{\partial \nu }\frac{a^{-\nu }}{J\left(s,\nu \right)}{|}_{\nu =0}=sJ\left(s,0\right)\frac{-\log\left(a\right)a^{-\nu }J\left(s,\nu \right)-a^{-\nu }\partial J\left(s,\nu \right)/\partial \nu }{J{\left(s,\nu \right)}^{2}}{|}_{\nu =0}=sJ\left(s,0\right)\left(\frac{-\log\left(a\right)}{J\left(s,0\right)}+\frac{\int_{\Theta }M\left(\theta \right)e^{s^{\prime }\theta }d\theta }{J{\left(s,0\right)}^{2}}\right)=s\left(\frac{1}{J\left(s,0\right)}\int_{\Theta }M\left(\theta \right)e^{s^{\prime }\theta }d\theta -\log\left(a\right)\right)=\frac{s}{J\left(s,0\right)}\int_{\Theta }\left(M\left(\theta \right)-\log\left(a\right)\right)e^{s^{\prime }\theta }d\theta \text{.}$$

Note that this can also be written as

$$E\left(s,0\right)=\frac{s}{J\left(s,0\right)}\int_{\left\|\theta \right\|<\rho }\log\left(\frac{c\left({\left\|\theta \right\|}^{2}\right)}{c\left(\rho^{2}-\right)}\right)e^{s^{\prime }\theta }d\theta$$

where the integral is always negative. □

Remark More generally, for θ∈int(Θ)

$$\nabla \left(e^{s^{\prime }\theta -\nu M\left(\theta \right)}g\left(\theta \right)\right)=\left(\left(s-\nu \nabla M\left(\theta \right)\right)g\left(\theta \right)+\nabla g\left(\theta \right)\right)e^{s^{\prime }\theta -\nu M\left(\theta \right)}\text{,}$$

so that if θ follows a proper conjugate prior with parameters s and ν,

$$E\left(\left(\nu \nabla M\left(\theta \right)-s\right)g\left(\theta \right)\right)=E\left(\nabla g\left(\theta \right)\right)-\frac{1}{J\left(s,\nu \right)}\int_{\Theta }\nabla \left(e^{s^{\prime }\theta -\nu M\left(\theta \right)}g\left(\theta \right)\right)d\theta \text{.}$$

This is used by Chou [2] to show if ν>0 and Θ is open (so that $e^{s^{\prime }\theta -\nu M\left(\theta \right)}$ tends to 0 as θ approaches the boundary of Θ) and g satisfies certain integrability conditions, the integral vanishes so that E((ν∇M(θ)−s)g(θ))=E(∇g(θ)). If Θ is not open, the integral does not necessarily vanish. In the spherical case, if g is radial (i.e.,  $g\left(\theta \right)=\phi \left({\left\|\theta \right\|}^{2}\right)$), proceeding as above one can show that the integral equals $c{\left(\rho^{2}-\right)}^{-\nu }\phi \left(\rho^{2}\right)sJ\left(s,0\right)$.

If θ follows a proper conjugate prior with parameters s and ν, in the regular case its posterior expectation is given by

$$E\left(\nabla M\left(\theta \right)|x\right)=\frac{s+x}{\nu +1}$$

provided that ν+1>0, and hence is linear in x. In the non-regular case, the following result shows that posterior linear expectation never occurs.

Theorem 5 *Let* η *be the spherical probability distribution on* $R^{d}$ *generated by* $\eta_{R}$*, with moment generator* c*,* $0<\rho^{2}<\infty$*the radius of convergence of* c*, and* $c\left(\rho^{2}-\right)<\infty$ *. Suppose* θ *follows a proper conjugate prior with parameters* s *and* ν *for the NEF through* η *. Then linearity of posterior expectation does not hold.*

Proof From Theorem 4, if θ follows a conjugate prior with parameters s and ν, E(∇M(θ))=E(s,ν) is of the form sγ(s,ν) where γ is scalar. Linearity of posterior expectation thus holds if and only if there are α∈R and $b\in R^{d}$ such that

E(s+x,ν+1)=(s+x)γ(s+x,ν+1)=αx+b

for all x in supp(η), the support of η. Rewrite this as

(s+x)(γ(s+x,ν+1)−α)=b−αs.

If the right hand side were non-zero, necessarily γ(s+x,ν+1)≠α and hence

$$x=\frac{1}{\gamma \left(s+x,\nu +1\right)-\alpha }\left(b-\alpha s\right)-s\text{,}$$

so that supp(η) were contained in the line through −s with direction b−αs, which is impossible as η is non-degenerate and spherical (so that its support certainly contains a hypersphere with positive radius). Thus, the right hand side must be zero, and hence γ(s+x,ν+1)=α for all x in supp(η) for which s+x≠0. If x=−s is in supp(η), it can be approximated by elements with the same length different from −s; thus, as γ is clearly continuous in its first argument, γ(s+x,ν+1)=α for all x in supp(η).

To simplify notations, take $c\left(\rho^{2}\right)=c\left(\rho^{2}-\right)$, so that $r\mapsto c{\left(r^{2}\right)}^{-\nu }$ is continuous on [0,ρ] for all ν, and $r\mapsto \log\left(c\left(r^{2}\right)/c\left(\rho^{2}\right)\right)$ is continuous on [0,ρ]. If h is continuous on $\left[0,\rho^{2}\right]$, transformation to polar coordinates gives

$$\int_{\Theta }e^{s^{\prime }\theta }h\left({\left\|\theta \right\|}^{2}\right)d\theta =\int_{0}^{\rho }\int_{S^{d-1}}h\left(r^{2}\right)e^{{\left(rs\right)}^{\prime }u}a_{d}r^{d-1}drd\eta_{U}\left(u\right)=a_{d}\int_{0}^{\rho }h\left(r^{2}\right){}_{0}F_{1}\left(;d/2;r^{2}{\left\|s\right\|}^{2}/4\right)r^{d-1}dr=a_{d}\int_{0}^{\rho }h\left(r^{2}\right)\left(\overset{\infty }{\underset{n=0}{\sum }}c_{n}{\left(r^{2}{\left\|s\right\|}^{2}\right)}^{n}\right)r^{d-1}dr=a_{d}\overset{\infty }{\underset{n=0}{\sum }}c_{n}\left(\int_{0}^{\rho }h\left(r^{2}\right)r^{2n+d-1}dr\right){\left\|s\right\|}^{2n}\text{,}$$

all rearrangements justified by absolute convergence. As h is bounded on $\left[0,\rho^{2}\right]$, the series

$$f_{h}\left(z\right)=a_{d}\overset{\infty }{\underset{n=0}{\sum }}c_{n}\left(\int_{0}^{\rho }h\left(r^{2}\right)r^{2n+d-1}dr\right)z^{n}$$

converges for all z, and hence defines an entire function.

If ν+1≠0, γ(s+x,ν+1)=α for all x in supp(η) is equivalent to Δ(x)=J(s+x,0)−δJ(s+x,ν+1)=0 for all x in supp(η), where $\delta =c{\left(\rho^{2}\right)}^{\nu +1}\left(1-\alpha \left(\nu +1\right)\right)$. Now $J\left(s,\nu \right)=f_{h}\left({\left\|s\right\|}^{2}\right)$ for $h\left(r^{2}\right)=c{\left(r^{2}\right)}^{-\nu }$ and hence $\Delta \left(x\right)=f_{h}\left({\left\|s+x\right\|}^{2}\right)$ for $h\left(r^{2}\right)=1-\delta c{\left(r^{2}\right)}^{-\left(\nu +1\right)}$. If ν+1=0, γ(s+x,0)=α for all x in supp(η) is equivalent to $\Delta \left(x\right)=\int_{\Theta }\log\left(c\left({\left\|\theta \right\|}^{2}\right)/c\left(\rho^{2}\right)\right)e^{{\left(s+x\right)}^{\prime }\theta }d\theta -\alpha J\left(s+x,0\right)=f_{h}\left({\left\|s+x\right\|}^{2}\right)=0$ for $h\left(r^{2}\right)=\log\left(c\left(r^{2}\right)/c\left(\rho^{2}\right)\right)-\alpha$. In both cases, h is monotone and does not vanish identically. As ρ<∞ is only possible if η has unbounded support, $f_{h}\left({\left\|s+x\right\|}^{2}\right)=0$ for all x∈supp(η) implies that there is a sequence $\sigma_{k}$ of non-negative reals with $\lim_{k}\sigma_{k}\to \infty$ and $f_{h}\left(\sigma_{k}\right)=0$, and the proof will be completed by showing that this is not possible.

Let us first show that we can always find an $n_{0}$ such that the coefficients $\gamma_{n}\left(h\right)=\int_{0}^{\rho }h\left(r^{2}\right)r^{2n+d-1}dr$ all have the same non-zero sign for $n\geq n_{0}$. This is trivial if h is non-negative or non-positive. If h changes from negative to positive, there exists 0<β<ρ such that $h\left(\beta^{2}\right)>0$. Hence,

$$\gamma_{n}\left(h\right)=\int_{0}^{\beta }h\left(r^{2}\right)r^{2n+d-1}dr+\int_{\beta }^{\rho }h\left(r^{2}\right)r^{2n+d-1}dr\geq h\left(0\right)\int_{0}^{\beta }r^{2n+d-1}dr+h\left(\beta^{2}\right)\int_{\beta }^{\rho }r^{2n+d-1}dr=\frac{h\left(\beta^{2}\right)\rho^{2n+d}}{2n+d}\left(1+\left(\frac{h\left(0\right)}{h\left(\beta^{2}\right)}-1\right){\left(\frac{\beta }{\rho }\right)}^{2n+d}\right)\text{,}$$

where the term in parentheses tends to one as n→∞. Hence, $\gamma_{n}\left(h\right)>0$ for all n sufficiently large. Similarly, if h changes from positive to negative, $\gamma_{n}\left(h\right)<0$ for all n sufficiently large.

If $\gamma_{n}\left(h\right)>0$ for all $n\geq n_{0}$ and σ>0,

$$f_{h}\left(\sigma \right)=a_{d}\overset{\infty }{\underset{n=0}{\sum }}c_{n}\gamma_{n}\left(h\right)\sigma^{n}\geq a_{d}\overset{n_{0}}{\underset{n=0}{\sum }}c_{n}\gamma_{n}\left(h\right)\sigma^{n}=a_{d}c_{n_{0}}\gamma_{n_{0}}\left(h\right)\sigma^{n_{0}}\left(1+\underset{0\leq n<n_{0}}{\sum }\frac{c_{n}\gamma_{n}\left(h\right)}{c_{n_{0}}\gamma_{n_{0}}\left(h\right)}\sigma^{n-n_{0}}\right)$$

where the term in parentheses tends to one as σ→∞. Hence, $f_{h}\left(\sigma \right)>0$ for all σ sufficiently large and positive. Similarly, if $\gamma_{n}\left(h\right)<0$ for all n sufficiently large, we have $f_{h}\left(\sigma \right)<0$ for all σ sufficiently large and positive, and the proof is complete. □

Remark The results of Theorems 3 and 4 in Diaconis and Ylvisaker [4] and Chou [3] characterize the canonical priors as those achieving linear posterior expectation, assuming that Θ is open. Theorem 5 shows that if Θ is closed, then the canonical priors never achieve linear posterior expectation in the case of NEFs through spherical probability distributions. This leaves open the question whether linear posterior expectation could be achieved by other priors.

## 4 NEFs through elliptical probability distributions

Following Fang et al. [5, p. 31f], we say that a probability distribution η on $R^{d}$ is *elliptical* if for some k∈{1,…,d} there are $m\in R^{d}$, a k×d matrix A of rank k, and a spherical probability distribution $\eta_{S}$ on $R^{k}$ such that $\eta \left(B\right)=\eta_{S}\left(\left\{y:A^{\prime }y+m\in B\right\}\right)$ for all Borel sets B in $R^{d}$. In this section, we discuss how the results in the previous section can be generalized from NEFs through spherical distributions to NEFs through elliptical distributions.

We have

$$\int_{R^{d}}e^{\theta^{\prime }x}\;d\eta \left(x\right)=\int_{R^{k}}e^{\theta^{\prime }\left(A^{\prime }y+m\right)}\;d\eta_{S}\left(y\right)=e^{\theta^{\prime }m}\int_{R^{k}}e^{{\left(A\theta \right)}^{\prime }y}\;d\eta_{S}\left(y\right)\text{.}$$

Hence, with M and $M_{S}$ the cumulant generating functions of η and $\eta_{S}$, respectively, we have

$$M\left(\theta \right)=\theta^{\prime }m+M_{S}\left(A\theta \right)\text{.}$$

The NEF through η has densities given by

$$f\left(x|\theta \right)=e^{\theta^{\prime }x-M\left(\theta \right)}=e^{\theta^{\prime }\left(x-m\right)-M_{S}\left(A\theta \right)}\text{,}$$

natural parameter space $\Theta =\left\{\theta :M_{S}\left(A\theta \right)<\infty \right\}$, and standard conjugate distributions with densities

$$\pi \left(\theta |s,\nu \right)\propto e^{s^{\prime }\theta -\nu M\left(\theta \right)}=e^{{\left(s-\nu m\right)}^{\prime }\theta -\nu M_{S}\left(A\theta \right)}$$

with normalizing constants

$$J\left(s,\nu \right)=\int_{\Theta }e^{{\left(s-\nu m\right)}^{\prime }\theta -\nu M_{S}\left(A\theta \right)}\;d\theta$$

if proper (i.e.,  J(s,ν)<∞). If k<n, the null space of A must at least have dimension one, so that Θ is always unbounded and J(s,ν) is never finite. Thus, in what follows we only consider the case where k=d and hence A is invertible, in which case Θ is bounded if and only if the natural parameter space $\Theta_{S}$ of the NEF through $\eta_{S}$ is bounded, and

$$J\left(s,\nu \right)=\int_{\theta :M_{S}\left(A\theta \right)<\infty }e^{{\left(s-\nu m\right)}^{\prime }A^{-1}A\theta }e^{-\nu M_{S}\left(A\theta \right)}\;d\theta ={\left|\det\left(A\right)\right|}^{-1}\int_{\gamma :M_{S}\left(\gamma \right)<\infty }e^{{\left(s-\nu m\right)}^{\prime }A^{-1}\gamma }e^{-\nu M_{S}\left(\gamma \right)}\;d\gamma ={\left|\det\left(A\right)\right|}^{-1}J_{S}\left(\tau \left(A\right)\left(s-\nu m\right),\nu \right)$$

where $\tau \left(A\right)={\left(A^{-1}\right)}^{\prime }$. Thus, the set of parameters s and ν giving proper conjugate priors for the NEF through η can straightforwardly be obtained from the corresponding set for the NEF through $\eta_{S}$.

The above computations did not use the fact that $\eta_{S}$ is spherical. Actually doing so and writing $c_{S}$ for the moment generator of $\eta_{S}$, we have $M_{S}\left(A\theta \right)=c_{S}\left({\left\|A\theta \right\|}^{2}\right)=c_{S}\left(\theta^{\prime }A^{\prime }A\theta \right)$. Thus, M(θ) and hence also the distribution of η depend on A only through $\Sigma =A^{\prime }A$, and if k=d we can take $A=\Sigma^{1/2}$ as the symmetric root of Σ (in which case $\tau \left(A\right)=\Sigma^{-1/2}$).

If $c_{S}$ has finite radius of convergence $\rho_{S}$ (so that Θ and $\Theta_{S}$ are bounded), the above relation between J and $J_{S}$ implies that $\nu_{0}\left(\eta \right)=\nu_{0}\left(\eta_{S}\right)$ and hence can be characterized using Theorems 2 and 3.

For hyperparameters s and ν giving a proper conjugate prior for the NEF through η, write

E(s,ν)=E(∇M(θ)|s,ν)

(where the expectation is taken with respect to this prior), and write $E_{S}$ for the corresponding quantity for $\eta_{S}$. As clearly $\nabla M\left(\theta \right)=m+A^{\prime }\nabla M_{S}\left(A\theta \right)$ on int(Θ), we have

$$E\left(s,\nu \right)=\frac{1}{J\left(s,\nu \right)}\int_{\Theta }\nabla M\left(\theta \right)e^{s^{\prime }\theta -\nu M\left(\theta \right)}d\theta =m+\frac{1}{J\left(s,\nu \right)}\int_{\Theta }A^{\prime }\nabla M_{S}\left(A\theta \right)e^{{\left(s-\nu m\right)}^{\prime }A^{-1}A\theta }e^{-\nu M_{S}\left(A\theta \right)}\;d\theta =m+A^{\prime }E_{S}\left(\tau \left(A\right)\left(s-\nu m\right),\nu \right)\text{,}$$

where in turn $E_{S}$ can be obtained using Theorem 4. In particular, in the regular case (a), we have $E_{S}\left(s,\nu \right)=s/\nu$ for ν>0, and hence also

$$E\left(s,\nu \right)=m+A^{\prime }\tau \left(A\right)\left(s-\nu m\right)/\nu =m+\left(s-\nu m\right)/\nu =s/\nu \text{.}$$

Finally, let us show that if the NEF through η is non-regular (i.e., if $\Theta =\left\{\theta :\theta^{\prime }\Sigma \theta \leq \rho_{S}^{2}\right\}$), posterior linear expectation never occurs. As the proof of Theorem 5 shows, linearity of posterior expectation is equivalent so the existence of α and b such that

$$\alpha x+b=E\left(s+x,\nu +1\right)=m+A^{\prime }E_{S}\left(\tau \left(A\right)\left(s+x-\left(\nu +1\right)m\right),\nu \right)$$

or equivalently,

$$E_{S}\left(\tau \left(A\right)\left(s+x-\left(\nu +1\right)m\right),\nu \right)=\tau \left(A\right)\left(\alpha x+\left(b-m\right)\right)$$

for all x in the support of η. But clearly, x is in the support of η if and only if y=τ(A)(x−m) is in the support of $\eta_{S}$, so that the above is equivalent to having

$$E_{S}\left(\tau \left(A\right)\left(s-\nu m\right)+y,\nu +1\right)=\alpha y+\tau \left(A\right)\left(b-m+\alpha m\right)$$

for all y in the support of $\eta_{S}$. This in turn is equivalent to linearity of posterior expectation for the conjugate prior for the NEF through $\eta_{S}$ with parameters τ(A)(s−νm) and ν, which by Theorem 5 cannot hold if this family (and equivalently the NEF through η) is non-regular.
